# Histological spectrum of pulmonary manifestations in kidney transplant recipients on sirolimus inclusive immunosuppressive regimens

**DOI:** 10.1186/1746-1596-7-25

**Published:** 2012-03-14

**Authors:** Sean Kirby, Anjali Satoskar, Sergey Brodsky, Amy Pope-Harman, David Nunley, Charles Hitchcock, Ronald Pelletier, Patrick Ross, Tibor Nadasdy, Konstantin Shilo

**Affiliations:** 1 Department of Pathology, The Ohio State University Medical Center, Columbus, Ohio, USA; 2 Department of Medicine, The Ohio State University Medical Center, Columbus, Ohio, USA; 3 Department of Surgery, The Ohio State University Medical Center, Columbus, Ohio, USA

**Keywords:** Kidney transplantation, Pulmonary neoplasia, Pulmonary hemorrhage, Mammalian target of rapamycin (mTOR) inhibitors, Sirolimus

## Abstract

**Background:**

After the introduction of novel effective immunosuppressive therapies, kidney transplantation became the treatment of choice for end stage renal disease. While these new therapies lead to better graft survival, they can also cause a variety of complications. Only small series or case reports describe pulmonary pathology in renal allograft recipients on mTOR inhibitor inclusive therapies. The goal of this study was to provide a systematic review of thoracic biopsies in kidney transplant recipients for possible association between a type of immunosuppressive regimen and pulmonary complications.

**Methods:**

A laboratory database search revealed 28 of 2140 renal allograft recipients (18 males and 10 females, 25 to 77 years old, mean age 53 years) who required a biopsy for respiratory symptoms. The histological features were correlated with clinical findings including immunosuppressive medications.

**Results:**

The incidence of neoplasia on lung biopsy was 0.4% (9 cases), which included 3 squamous cell carcinomas, 2 adenocarcinomas, 1 diffuse large B-cell lymphoma, 1 lymphomatoid granulomatosis, and 2 post transplant B-cell lymphoproliferative disorders. Diffuse parenchymal lung disease was identified in 0.4% (9 cases), and included 5 cases of pulmonary hemorrhage, 3 cases of organizing pneumonia and 1 case of pulmonary alveolar proteinosis. Five (0.2%) cases showed histological features indicative of a localized infectious process. Patients on sirolimus had neoplasia less frequently than patients on other immunosuppressive combinations (12.5% vs. 58.3%, *p *= 0.03). Lung biopsies in 4 of 5 patients with clinically suspected sirolimus toxicity revealed pulmonary hemorrhage as the sole histological finding or in combination with other patterns.

**Conclusions:**

Our study documents a spectrum of neoplastic and non-neoplastic lesions in renal allograft recipients on current immunosuppressive therapies. Sirolimus inclusive regimens are associated with increased risk of pulmonary toxicity but may be beneficial in cases of posttransplant neoplasia.

**Virtual Slides:**

The virtual slide(s) for this article can be found here: http://www.diagnosticpathology.diagnomx.eu/vs/3320012126569395.

## Background

Kidney transplantation is considered the treatment of choice for end stage renal disease (ESRD), which is in part due to availability of more effective immunosuppressive regimens. The mammalian target of rapamycin (mTOR) inhibitors, rapamycin, also known as sirolimus, and everolimus, have been recently widely utilized in immunosuppressive regimens providing adequate immunosuppression and avoiding nephrotoxic side effects of calcineurin inhibitor therapy [1-4]. However, prolonged graft survival leads to increased incidence of complications related to both immunosuppression and drug toxicity. Drug induced immunosuppression halts tumor surveillance leading to an increase in tumor development. Epidemiologic studies show that posttransplant lymphoproliferative disorder (PTLD) and skin cancers increased most dramatically following kidney transplantation [5-8]. In addition to immunosuppression, sirolimus exhibits antineoplastic properties in vivo [9] and newer rapamycin analogs have been evaluated in clinical trials for treatment of renal cell carcinoma [10]. Clinical experience with these medications is limited; but it has been shown to cause regression of PTLD and Kaposi sarcoma [11-14]. The incidence of pulmonary toxicity in patients on mTOR inhibitors has been reported to be up to 11% [15,16]. Risk factors for the development of sirolimus-associated pneumonitis include higher dose, greater trough levels and older age [17]. While the contribution of other causes in the setting of mTOR inhibitor induced immunosuppression is difficult to separate from direct drug toxicity, a range of pulmonary histopathologic changes has been suggested as manifestations of drug toxicity. Depending on biopsy modality these include descriptive diagnoses or better-defined histological patterns such as organizing pneumonia and diffuse alveolar hemorrhage [15,16,18-20]. Pulmonary hemorrhage has been reported as a sole histological finding [21] but also in combination with others [18]. Other rare pulmonary manifestations include pulmonary alveolar proteinosis [22], desquamative interstitial pneumonitis [23], hypersensitivity pneumonitis [24], necrotizing granulomas and vasculitis [25], diffuse alveolar damage [26] and non-necrotizing granulomas [19]. Since the reported histological manifestations are not specific for sirolimus toxicity, drug discontinuation with or without steroid therapy is the mainstay of treatment in suspected cases and typically leads to resolution of symptoms within 2 to 4 months [18]. To the best of our knowledge, only small series or case reports describe pulmonary pathology in renal allograft recipients and the concept of sirolimus-associated pulmonary complications is still evolving. Therefore, the goals of this study were to provide a systematic review of pulmonary histological findings in the setting of kidney transplantation and elucidate the possible contribution of the current immunosuppressive regimens to the spectrum of the observed histological changes.

## Material and methods

A laboratory information system database search from January 2002 to September 2010 revealed 28 renal allograft recipients who required a lung biopsy for respiratory symptoms. In total, 42 biopsies were performed, including 8 (19%) video-assisted thoracoscopic biopsies, 28 (67%) endobronchial biopsies, 5 (12%) needle biopsies, and 1 (2%) mediastinal lymph node biopsy. Sixteen (38%) biopsies were nondiagnostic. Biopsies were considered non-diagnostic if they had limited tissue or minimal nonspecific histological changes such as chronic inflammation or focal interstitial fibrosis. Hematoxylin-eosin (H&E) stained slides (range, 1-19; mean, 3 slides) and special stains for microorganisms including Gomori methenamine silver, Ziehl-Neelsen and gram stains, performed on formalin-fixed, paraffinembedded tissue were re-examined. The radiology studies including chest computed tomography (CT) were reviewed in respect to localized versus diffuse changes. Localized lesions included nodules or masses while the diffuse lesions were comprised of diffuse or patchy bilateral ground glass opacities, reticular densities, bilateral consolidations, mosaic attenuation, and traction bronchiectasis. Retrospective analysis of electronic clinical records and correlation with histological findings and type of immunosuppressive therapy were performed. The electronic medical records were reviewed with special attention for evidence of systemic diseases and infectious complications including microbiological cultures and serologies for viral and fungal pathogens. Review of immunosuppressive regimens included an active list of medications pre-and post-lung biopsy. Clinical and pathological findings were analyzed utilizing mean ± SD for continuous variables, and number or percentage for categorical variables. Comparisons were performed using Yates' chi-square test for categorical variables, and the two-sample *t *test for continuous variables. The study was approved by The Ohio State Biomedical Sciences Institutional Review Board in compliance with Health Insurance Portability and Accountability Act regulations.

## Results

### Clinicopathological findings in patients with kidney transplant

The main clinical and pathological findings are summarized in Table 1. Twenty-eight of 2140 (1.3%) kidney transplant recipients over the period of 105 months underwent a lung biopsy for pulmonary symptoms. They included 18 males and 10 females with an age range from 25 to 77 years old (mean age of 53 years). The time from kidney transplantation to lung biopsy ranged from 4 to 345 months (mean 81 months). In 19 cases (68%) the biopsies were performed to obtain tissue diagnosis for localized lesions, and in 9 cases (32%) for diffuse lesions. The majority of patients (18/28 or 64%) received a deceased donor kidney. Eight patients (29%) received kidneys from living related donors; two patients (7%) received kidneys from living unrelated donors. One patient received three transplants, including two cadaveric and one living related. Twenty-five (89%) patients had kidney-only transplant, while three patients (11%) had a combined kidney and pancreas transplantation. The most common cause of ESRD was diabetic nephropathy (10/28; 36%). Other causes included hypertension (2/28; 7%), polycystic kidney disease (2/28; 7%), glomerulonephritis (4/28; 14%), granulomatosis with polyangiitis (Wegner's granulomatosis) (1/28; 4%), IgA nephropathy (1/28; 4%), chronic reflux (1/28; 4%), p-ANCA-positive microscopic polyangiitis (1/28; 4%), and interstitial nephritis (1/28; 4%).

**Table 1:** Clinical pathological findings in kidney transplant recipients

	Age, years	Gender	Kidney disease	Transplant type	Time, mos	Sirolimus trough levels, mean(range), ng/ml	Other immuno-suppressants	Clinical radiological presentation	Biopsy type	Pathological findings
1*	49	M**	DM1	K/Cad	11	11.7(6.2-18.7)	C/P	Bilateral mass-like consolidations	EB	ND

2	25	F	DM1	K/Cad	20		C/MY/D	Patchy bilateral consolidations	EB	BCLPD

3	71	M	PCKD	K/Cad	36		C/D	Mediastinal lymphadenopathy	EB LNB	DLBCL

4	58	F	DM2	K/Cad	5	8.8(4.0-17.1)	C/P	Respiratory failure Diffuse ground glass opacities	EB THB	Hemorrhage

5	47	F	HTN	K/Cad	210		C/MY/P	Respiratory failure, loculated pneumonia	EB	Necrosis Fibrin

6	72	M	DM2	K/Cad	20	4.8(2.0-8.2)	C/P	Spiculated lung lesion	NB	SQC

7	77	M		K/Cad	116		C/D	Pulmonary nodule	EB	SQC

8	71	M	pANCA	K/Cad	10	15.1	C/P	Right lower lobe mass	EB	ND

								Diffuse ground glass opacities		

9	48	F	NA	K/Cad	32	NA	MY/P	Left lower lobe nodule	EB	Necrosis Fibrin

10	39	M	GN	K/Cad	80	10.2(8.1-11.8)	C/P	Respiratory failure, diffuse ground glass opacities with crazy paving pattern	THB EB	Hemorrhage PAP OP

11	45	F	NA	K/Cad, LR	345		C/P	Collapsed lung, pneumonia	EB	OP

12	58	F	HTN	K/Cad	24	19.7	C/P	Respiratory failure, diffuse ground glass opacities	EB THB	Hemorrhage

13	44	F	DM2	K/LR	36	5.3(3.2-6.9)	C/P	Mediastinal lymphadenopathy, patchy ground glass opacities	EB	ND

14	46	M	WG	K/LR	175		C/MY/P	Diffuse ground glass opacities, lung nodules	EB NB	Hemorrhage Capillaritis

15	49	F	DM	K	73	8.1(4.3-11.6)	C/P	Bilateral ground glass opacification with right lower lobe consolidation	EB	OP

16	34	F	DM1	KP/Cad	19	4.2(2.4-6.7)	C/P	Respiratory failure, diffuse ground glass opacities	THB	DAD Hemorrhage

17	43	M	DM1	KP	41	4.4	C/P	Nodular right lower lobe infiltrate	EB	ND

18	69	M	DM2	K/LU	4	11.0(5.0-14.9)	C/P	Bilateral lung nodules and mediastinal lymphadenopathy	NB	BCLPD

19	71	M	IgA	K/LR	114		C/P	Dyspnea, diffuse ground glass opacities	EB	PAP

20	60	M	GN	K/LR	89		C/MY/D	Lung nodule	EB	ND

21	65	M		K/LR	41	6.8(2.8-12.0)	MA/P	Bilateral hypermetabolic lung nodules	THB	PJ granulomas

22	60	M	GN	K/LR	66		C/P	Lung mass	THB	ADC

23	52	M	DM1	KP/Cad	73		C/P	Lung mass	EB	SQC

24	34	M	GN	K/Cad	38	10.2(6.1-14.3)	C/P	Bilateral ground glass opacities Loculated right sided pleural effusion	EB	Fibrin Necrosis

25	36	F	NA	K	180		C/P	Bilateral lung nodules, lymphadenopathy	THB NB	LYG

26	62	M	IN	K/LR	115		C/P	Lung mass	NB	ADC

27	53	M	PCKD	K/LU	61	2.0	MA/P	Lung nodule, pleural effusion	EB	ND

28	32	M	CR	K/LU	220	8.6(4.4-13.2)	C/MA/P	Dyspnea, patchy ground glass opacities	EB	OP

(*) - the cases are arranged in chronological order; (**)-*M *male; *F *female; *DM *diabetes mellitus; *PCKD *polycystic kidney disease; *HTN *hypertension; *p-ANCA *p-ANCA vasculitis; *GN *glomerulonephritis; *WG *Wegner's granulomatosis; *IgA *IgA nephropathy; *IN *interstitial nephritis; *CR *chronic reflux; *K *kidney transplant; *KP *kidney and pancreas transplant; *Cad *cadaveric kidney; *LR *living related kidney; *LU *living unrelated kidney; *EB *endobronchial biopsy; *THB *thoracoscopic biopsy; *NB *needle biopsy; *LNB *lymph node biopsy; *C *cyclosporine; *P *prednisone; *D *dexamethasone; *MY *mycophenolate mofetil; *MA *mycophenolic acid; *ND *not diagnostic; *SQC *squamous cell carcinoma; *ADC *adenocarcinoma; *BCLPD *B-cell lymphoproliferative disorder; *DLBCL *diffuse large B-cell lymphoma; *LYG *lymphomatoid granulomatosis; *OP *organizing pneumonia; *DAD *diffuse alveolar damage; *PJ *Pneumocystis jiroveci; *PAP *pulmonary alveolar proteinosis; *NA *not available

**Figure 1:**
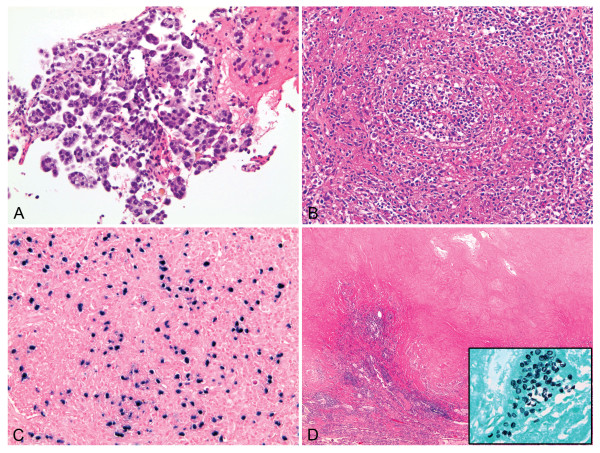
**Neoplastic and non-neoplastic localized lesions in renal transplant recipients**. (A) adenocarcinoma with predominantly micropapillary pattern (case 22, hematoxylin-eosin, original magnification x200); (B) lymphomatoid granulomatosis showing angiocentric proliferation of atypical lymphoid cells associated with Epstein-Bar virus (C), (case 25, hematoxylin-eosin and colorimetric in situ hybridization, original magnification x200 and x400, respectively); necrotizing granuloma (D) associated with *Pneumocystis jiroveci *(case 21, hematoxylin-eosin and Gomori methenamine silver, inset, original magnification x100 and x600, respectively).

**Figure 2:**
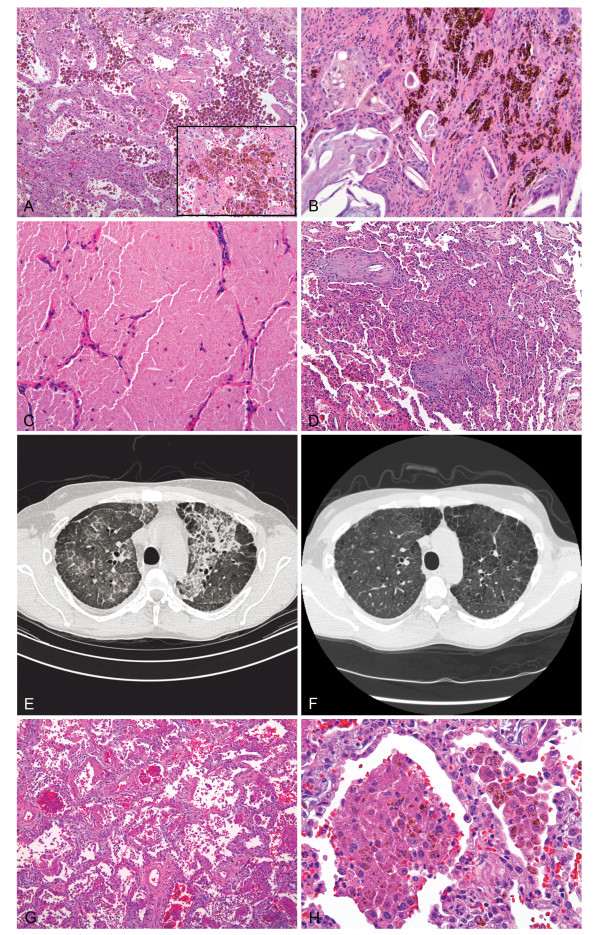
**Pathological findings in patients with sirolimus toxicity**. In case 4, open lung biopsy shows diffuse alveolar hemorrhage represented by collections of hemosiderin laden alveolar macrophages (A, inset) and occasional hemosiderin granules within interstitium, (hematoxylin-eosin, original magnification x100 and x400, respectively). In case 10, open lung biopsy shows pulmonary hemorrhage in association with pulmonary alveolar proteinosis and organizing pneumonia: there are areas with hemosiderin deposition within interstitium (B) and cholesterol granulomas, finely granular proteinaceous material with cholesterol clefting (C) and foci of organizing pneumonia (D) in adjacent alveolar parenchyma, (hematoxylin-eosin, original magnification x100, x200 and × 40, respectively). Computed tomography of the chest at the time of open lung biopsy shows diffuse ground glass and "crazy pavement" opacities (E). Follow up computed tomography in 8 months (F) shows marked decrease in alveolar opacities; both scans are at the level of aortic arch for comparison. In case 16, open lung biopsy shows a combination of diffuse alveolar damage and pulmonary hemorrhage: there are edematous alveolar septae lined by hyaline membranes (G) and hemosiderin-laden macrophages (H) within alveolar spaces (H&E, original magnification x200 and x400, respectively).

Neoplasia on lung biopsy was identified in 9 (0.4%) of 2140 kidney transplant recipients. Among 9 cases there were 5 non-small cell carcinomas and 4 PTLD. Non-small cell carcinomas included 3 cases of squamous cell carcinoma and 2 cases of adenocarcinoma (Figure 1A). PTLD included 1 case of diffuse large B-cell lymphoma, 1 case of lymphomatoid granulomatosis (Figure 1B-C) and 2 cases of post transplant B-cell lymphoproliferative disorders. Diffuse parenchymal lung disease was identified in 9 (0.4%) cases. In 2 cases, pulmonary hemorrhage (PH) was the sole histological finding. In 1 case PH was associated with capillaritis. In 1 case PH was associated with pulmonary alveolar proteinosis (PAP) and in 1 case with diffuse alveolar damage (DAD). PH associated with capillaritis was documented in a patient with WG and was considered a pulmonary manifestation of the disease. Organizing pneumonia (OP) as the main histological finding was identified in 3 cases and PAP was identified in 1 case. Five (0.2%) cases showed histological features indicative of an infectious process including tissue necrosis, necrotic cellular debris, acute inflammation, and granulomas. In 1 of 5 cases, granulomatous inflammation was associated with *Pneumocystis jiroveci *(Figure 1D). Lung biopsy showed minimal findings in 5 (0.2%) patients.

### Clinicopathological findings in patients on sirolimus

All patients with a lung tissue diagnosis received combination immunosuppressive therapy. The immunosuppressive regimen of 16 of 28 (57%) patients included sirolimus (Table 2). Other immunosuppressants were comprised of cyclosporine (25/28; 89%), prednisone (23/28; 82%), dexamethasone (4/28; 14%), mycophenolate mofetil (6/28; 21%), and mycophenolic acid (2/28; 7%). The groups of patients receiving sirolimus versus other immunosuppressive medications were of similar age and gender (Table 2). However, the mean time from the transplant to lung biopsy of patients on sirolimus was shorter (44.7 ± 52.04 vs. 128.3 ± 89.32, *p *= 0.01). Patients on sirolimus less frequently than patients on other immunosuppressants had neoplasia (12.5% vs. 58.3%, *p *= 0.03). Tumors in patients on sirolimus included 1 squamous cell carcinoma and 1 post transplant B-cell lymphoproliferative disorder. Tumors in the non-sirolimus group included 2 adenocarcinomas, 2 squamous cell carcinomas, 1 posttransplant B-cell lymphoproliferative disorders, 1 diffuse large B-cell lymphoma and 1 lymphomatoid granulomatosis. Patients on sirolimus had a tendency to have diffuse parenchymal lung disease (37.5% vs. 25.0%), including hemorrhage (25.0% vs.8.3%). The results, although suggestive of an association, did not achieve statistical significance.

**Table 2:** Clinicopathological findings in patients on sirolimus versus other immunosuppressive therapy

	Sirolimus n = 16	Non-Sirolimus n = 12	P, value
Age, mean, years	51.1 ± 13.25	54.3 ± 15.39	ns

Gender, M*	10 (62.5)	8 (66.7)	ns

F	6 (37.5)	4 (33.3)	ns

Time to lung biopsy, mean, months	44.7 ± 52.04	128.3 ± 89.32	0.01

Neoplasia, total, n (%)	2 (12.5)	7 (58.3)	0.03

Carcinoma, n (%)	1 (6.25)	4 (33.3)	ns

Lymphoproliferative disorders, n (%)	1 (6.25)	3 (25.0)	ns

Diffuse parenchymal lung disease, n (%)	6 (37.5)	3 (25.0)	ns

PH, n (%)	2 (12.5)	1 (8.3)	ns

PH and DAD, n (%)	1 (6.25)	0	-

PH and PAP, OP, n (%)	1 (6.25)	0	-

OP, n (%)	2 (12.5)	1 (8.3)	ns

PAP, n (%)	0	1 (8.3)	-

Necrosis/infectious granulomas, n (%)	4 (25.0)	1 (8.3)	ns

Minimal histological findings, n (%)	4 (25.0)	1 (8.3)	ns

(*) -*M *male; *F *female; *PH *pulmonary hemorrhage; *DAD *diffuse alveolar damage; *PAP *pulmonary alveolar proteinosis; *OP *organizing pneumonia; ns, not statistically significant

### Sirolimus toxicity and response to treatment modification

Sirolimus toxicity was suspected clinically in 5 (31.3%) of 16 patients. Their mean trough sirolimus levels were not statistically different from the other 10 cases on sirolimus (10.3 ± 5.72 ng/ml vs. 7.6 ± 3.56 ng/ml). Among these 5 cases, lung biopsies in 4 revealed pulmonary hemorrhage and OP, in 1. All 5 patients showed clinical and radiological improvement after the drug discontinuation. Their clinical course and response to treatment modification is detailed below.

In case 4, a 54 year old woman was admitted with recurrent shortness of breath for the fourth time in the five months following kidney transplant. The patient had a past medical history of chronic obstructive pulmonary disease and congestive heart failure with basic oxygen requirements of 3-4 l via nasal cannula. On her current admission she was in severe respiratory failure, requiring mechanical ventilation. Her trough sirolimus levels following transplantation were within range from 4.0 to 17.1, (mean, 8.8), normal 3-20 ng/ml. A chest CT on admission showed diffuse ground glass opacities and pleural effusions. Endobronchial biopsy was nondiagnostic, while a subsequent open lung biopsy showed collections of hemosiderin laden macrophages occupying alveolar spaces as well as hemosiderin granules within interstitium (Figure 2A). Evaluation for infectious organisms and vasculitis was negative. Since treatment for infection did not produce any significant improvement, sirolimus toxicity was suspected and sirolimus was discontinued. The patient returned to baseline respiratory status (3-4 l of oxygen) with improvements in bilateral opacities radiologically within 6 months. Following discharge, the patient required a single readmission for respiratory symptoms over the subsequent 33 months. At that time she was admitted for respiratory failure and subsequently expired. Postmortem examination revealed extensive hemosiderin deposition and a left upper lobe adenocarcinoma.

Case 10 is that of a 39-year-old African-American male, 80 months post kidney transplantation, hospitalized for increasing shortness of breath with increasing creatinine levels. His progressive hypoxia resulted in intubation. A chest CT showed diffuse bilateral ground glass opacities, "crazy pavement" pattern and focal nodular consolidation. His trough sirolimus levels within 6 months prior to admission were 8.1-11.8, (mean, 10.2) normal, 3-20 ng/ml. Hemosiderin laden macrophages associated with rare cholesterol granulomas were seen on transbronchial biopsy. His respiratory status continued to deteriorate and open lung biopsy was performed that showed alveolar and interstitial hemosiderin deposition accompanied by patchy organizing pneumonia, cholesterol granulomas and eosinophilic proteinaceous granular material (Figure 2B-D). The findings were compatible with a combination of PH and PAP. He underwent therapeutic bronchioloalveolar lavage and the decision was made to discontinue all immunosuppressive medications other than prednisone. During his nearly 2 month hospital stay, he was gradually weaned from ventilation support and his oxygen requirement at the time of discharge was 2 l of oxygen. Over the ensuing 8 months he showed clinical and radiological improvement (Figure 2E-F) on mycophenolate maintenance therapy. At 3.5 years of follow up, the patient was on maintenance mycophenolate therapy, had intermittent problems with recurrent lower respiratory tract infections, residual restrictive lung disease, and continued to use 2-3 l of oxygen via nasal cannula mostly with exercise.

Case 12 represents a 58-year old female who was hospitalized 24 months post kidney transplantation with shortness of breath, hypoxia, and renal failure. Her trough sirolimus level before admission was elevated to 19.7 ng/ml, normal 3-15 ng/ml. A bronchoscopic biopsy revealed occasional hemosiderin granules. Her symptoms did not improve and an open lung biopsy revealed histological evidence of PH with alveolar and interstitial hemosiderin deposition. Respiratory and blood cultures remained negative throughout the patient's stay. Sirolimus was stopped and over the following 4 months she showed significant clinical improvement despite continued baseline oxygen requirements.

Case 16 is that of a 34-year old female who was admitted in respiratory failure 17 months post kidney-pancreas transplant with two episodes of acute rejection. Her trough sirolimus levels were within range 2.4-6.7 ng/ml, mean 4.2 ng/ml, normal 3-15 ng/ml. Hemosiderin laden macrophages were present in the bronchioalveolar lavage fluid. Sirolimus toxicity was suspected and sirolimus was discontinued. Despite extensive work-up for infectious and rheumatologic etiologies and treatment with wide spectrum antibiotics she remained in respiratory failure. An open lung biopsy showed a combination of DAD and PH with hyaline membranes, architectural simplification with alveolar septal collapse, diffuse myxoid thickening, type 2 pneumocyte hyperplasia, and alveolar and interstitial hemosiderin (Figure 2G-H). The patient's treatment also included mechanical ventilation, volume and blood pressure control. After 2 months at a rehabilitation facility, her shortness of breath and cough were significantly improved and chest CT revealed resolution of the bilateral infiltrates. On further follow-up, the patient reported minimal respiratory symptoms.

Case 28 represents a 31-year-old male, 220 months post kidney transplantation, started to experience cough and dyspnea. He received a course of antibiotics for community-acquired pneumonia with no improvement. His trough sirolimus levels within previous 6 months were 4.4-13.2 ng/ml, (mean 8.6 ng/ml), normal 5-30 ng/ml. He underwent an extensive evaluation including a CT scan of the chest revealing extensive ground glass densities in a multifocal distribution. Evaluation for collagen vascular disease and infectious etiologies were negative, including a bronchoscopic examination with microbiological cultures. Transbronchial lung biopsy showed patchy organizing pneumonia and chronic inflammation. Since no systemic or infectious causes for his symptoms were found, sirolimus toxicity was suspected. Sirolimus was discontinued and he was given a course of oral corticosteroids. Over the following 2 months, his respiratory status improved significantly, with no reported shortness of breath, cough or wheezing and discontinuation of supplemental oxygen.

## Discussion

Kidney transplant recipients are known to be at increased risk for malignancy with up to 2 fold increased incidence rates for lung cancer and 20-fold increased incidence rates of PTLD [5]. Our retrospective review shows a wide spectrum of neoplastic and non-neoplastic lesions in the lungs of kidney transplant recipients on current immunosuppressive regimens. Among the neoplastic lesions there were 5 cases of non-small cell lung carcinoma and 4 cases of PTLD with incidence of approximately 0.2% each. The incidence of lung carcinoma in our series was similar to what was previously reported in other single center cohorts [27,28], exceeding the incidence seen in general population [5]. The incidence of PTLD was lower, which is likely due to the fact that our study examined only lung biopsies and therefore couldn't account for the PTLD involving other sites. PTLD reportedly affects 1.8% of patients with up to 50% of cases presenting with extranodal masses, including lung nodules [29-31].

When correlated to the type of immunosuppression, the frequency of neoplasia in patents on sirolimus was lower (12.5% vs. 58.3%, *p *= 0.03) relative to patients on other immunosuppressants. It is possible that the longer transplant to lung biopsy time in the non-sirolimus group may have contributed to increased tumor detection. Nevertheless, our findings are in agreement with studies supporting antineoplastic properties of mTOR inhibitors in pre-clinical testing [9,32,33] and clinical studies of patients with post transplant solid organ tumors [28,34-36], suggesting that sirolimus may be beneficial in preventing posttransplant malignancies.

Our study also demonstrates that pulmonary hemorrhage is a common histological finding in cases with clinically suspected sirolimus toxicity. In this cohort, it can be seen as the sole histological finding or in combination with other histological patterns including DAD and PAP. Based on their own experience and review of the literature, Pham and colleagues list pulmonary hemorrhage, organizing pneumonia, and lymphocytic pneumonitis among the most common histological patterns of sirolimus toxicity [18]. From a clinical management point of view, the histological diagnosis of pulmonary hemorrhage carries a task of elucidating its possible causes which generally include alveolar hemorrhage syndromes or secondary causes associated with infections, toxic inhalation, coagulopathies, renal failure with volume overload, and venous congestion due to heart disease to name a few [37]. In our series only one case with PH was linked to Wegener's granulomatosis. During their clinical course, the study patients experienced renal failure with wide fluctuation of serum creatinine levels; however, the renal function was generally well controlled, and none had clinically documented sustained fluid overload. In our cases pulmonary hemorrhage could not be explained by any other causes, and sirolimus discontinuation lead to the gradual clinical improvement. In case 4, the patient had congestive heart failure, which histologically can be associated with pulmonary hemosiderin deposition. Therefore, it is difficult to completely exclude the contribution of the patient's underlying heart disease to the observed histological findings. In that regard, this case is similar to that reported by Hashemi-Sadraei N and colleagues [38]. They described a renal transplant recipient who was on chronic anticoagulation therapy for a prosthetic aortic valve, and who developed pulmonary symptoms following initiation of sirolimus therapy. An open lung biopsy showed diffuse alveolar hemorrhage with fibrin deposits in the alveolar spaces and small bronchi. Even though the underlying disease, congestive heart failure or heart valve requiring anticoagulation therapy, may have contributed to the pulmonary hemosiderin deposition, in both of these cases the response to sirolimus discontinuation suggests a causative association between the drug and pulmonary hemorrhage.

Other histological patterns identified within a spectrum of the diffuse parenchymal lung disease in patients on sirolimus were OP, DAD, and PAP. The spectrum of etiologic considerations in OP includes infection, collagen vascular disease, toxic inhalation, aspiration pneumonia, hypersensitivity pneumonitis, and drug toxicity [39,40]. Additionally, OP can be a minor component of other interstitial lung diseases. OP is one of the relatively frequently reported manifestations of sirolimus toxicity [18]. We identified OP as a main histological feature in 3 cases; however, it was also observed as a minor component in other cases including cases of pulmonary hemorrhage and DAD. This could be a reason for some discrepancy between reported frequencies of OP, especially if a diagnosis is rendered on a small endobronchial or transbronchial biopsy [18]. DAD is a well recognized histological pattern known to be associated with drug toxicity [41]. Among tissue reactions associated with sirolimus toxicity, only one case of DAD has been documented in the literature. Manito and colleagues [26] reported a fatal course of DAD in a 52-year-old man heart transplant recipient following a loading-dose of sirolimus administration. We observed DAD in one patient on sirolimus (case 16) where an open lung biopsy revealed a combination of DAD and pulmonary hemorrhage. No infectious or systemic disease was documented with extensive clinical evaluation. Despite wide spectrum antibiotics coverage, the patient showed a protracted clinical course but gradually improved over 2 months after sirolimus discontinuation showing only minimal pulmonary symptoms.

PAP is a rare poorly understood disorder that is characterized by accumulation of lipoproteinaceous surfactant-like material within alveolar parenchyma. Impaired macrophage function due to antibodies to granulocyte macrophage-colony stimulating factor is thought to be a key mechanism in primary PAP. Macrophage dysfunction due to immunosuppression is considered as one among many other causes of secondary PAP. It has been linked to sirolimus toxicity in 2 previously reported cases [22,42]. PAP histology in our series was documented in both sirolimus (1 case) and non-sirolimus (1 case) groups, suggesting that this is a secondary immunosuppression related tissue reaction that is not directly related to sirolimus toxicity.

Sirolimus induced immunosuppression results from the inhibition of T and B lymphocyte proliferation through the same mechanisms as it inhibits cancer cell proliferation. These effects are thought to be mediated through the rapamycin-FKPB12 complex altering the mTOR signaling network which includes tumor suppressor genes (PTEN, LKB1, TSC1, and TSC2) and proto-oncogenes (PI3K, Akt, and eIF4E) [43]. While the exact mechanisms of sirolimus toxicity are not known, several hypotheses have emerged. Clinical improvement after sirolimus dose reduction provides evidence for a dose-dependant pulmonary toxicity. Clinically and radiologically documented pneumonitis in kidney transplant recipients has been reported to improve dramatically after sirolimus dose reduction and the maintenance of lower trough levels [44]. BAL fluid analysis in cases of the drug induced alveolitis showed a predominance of CD4-positive lymphocytes allowing the authors to suggest that a cell mediated autoimmune response may be one of the factors responsible for sirolimus induced pulmonary toxicity [44]. Furthermore, it has been speculated that the drug's high affinity for plasma proteins may render sirolimus immunogenic as a hapten eliciting cascade of T-cell mediated delayed type of hypersensitivity reaction [18]. These hypotheses appear to capture the state of current knowledge; however, detailed mechanisms of sirolimus toxicity and their relationship to the spectrum of histological patterns of parenchymal lung disease are yet to be elucidated.

## Conclusions

Our study documents that kidney transplant recipients show a range of pulmonary neoplastic and non-neoplastic lesions, which are likely associated with the type of immunosuppressive regimen. Current mTOR inhibitor inclusive regimens may account for decreased number of tumors in kidney transplant recipients but also carry a risk of pulmonary toxicity manifesting histologically by pulmonary hemorrhage, organizing pneumonia and other less common histological patterns.

## Competing interests

The authors declare that they have no competing interests.

## Authors' contributions

SK reviewed cases, analyzed findings and drafted the manuscript. AS, SB, AP-H, DN, RP, TN and KS designed the study, participated in data collection and edited the manuscript. CH and PR participated in data collection and edited the manuscript. KS also reviewed the cases, developed and approved the study protocol, revised and edited the manuscript. All authors have read and approved the final manuscript.
